# Father Damien.—A Life Given to the Lepers

**Published:** 1889-05-18

**Authors:** 


### FATHER DAMIEN.—A LIFE GIVEN TO THE LEPERS.

Never was a more enthusiastic yet sorrowful meeting than that held at the Princes' Hall, last Tuesday, to hear Mr. Edward Clifford give the testimony of an eye-witness as to the life and work of the hero-priest Father Damien. Only three days before had come the news that the sacrifice was consummated—that the life nobly given to the lepers was finally laid down in their cause. And with hearts shadowed with the thought of a strong soul fled, yet rejoicing in the thought of one more martyr who had won the crown, those who loved and reverenced Father Damien and the cause for which he worked gathered at Princes' Hall. Long ere three o'clock the room was crowded to excess, and visitors who only pleaded for standing room had to be refused. Some sketches of Molokai, done on the spot by Mr. Clifford, were on view, and represented scenery which might be that of Paradise. All the luxuriance of the tropics seemed to bless the islands, and the clear blue of the sea and soft green of the trees gave an appearance of richness to every sketch. Peculiar interest attached to the three sketches of Father Damien done from the life; they all showed a strong face, deeply lined. One bore a resemblance to the pictures of the Christ, but in the other two the tense, sorrowful expression and exaggeration of the features due to disease, hid this look. Father Damien is represented in a brown cassock, and in one sketch wears a straw sailor hat; it is easily seen that he was both man and priest, and that nothing small or petty ever troubled that strong sensible face.

The Church Army were in force at Princes' Hall, showing people to their seats, handing leaflets, etc.; some nine or ten of their nurses in neat black uniform, and with silver crosses in the front of their bonnets, were seated on the platform; and Mr. Carlile, the secretary of the Army, opened the proceedings by leading a hymn and offering a prayer. Mr. Clifford's address embodied much that is to be found in his article in the *Nineteenth Century*. Mr. Clifford himself is a very tall strong-looking man, with grey hair. He is treasurer to the Church Army and has worked well in its cause. His visit to Father Damien, which took place last year, was not his first mission. He found Father Damien a cheery active man, essentially practical, yet with the power of withdrawing into the inner life at all times of quiet and of rest. He lived in a bright little four-room house close to the church he was building, and which was the pride of his heart. He was helped in his labours by another father and two brothers of the order, and three Franciscan sisters also live and labour on the Molokai. The people of the island Mr. Clifford did not find so melancholy or so disfigured as he expected. They were rather bright and hospitable, with cheerful brown faces, and, when not much diseased, of muscular build. Father Damien informed Mr. Clifford he had no doubt that leprosy was contagious; that again and again helpers who had come to the island had contracted the disease. Women seem less liable to leprosy than men; one woman on the island had had four husbands who had all died lepers, yet she had never contracted the infection. The strong spirit of self-denial and the great religious fervour which animated Father Damien, were traits of his mother's character also. He was born in Belgium, and there trained for the priesthood, volunteering for missionary service ere he was twenty years old. There can be no doubt that, Protestants or Catholics, we can all learn a lesson from this noble and devout man, whose story reads like one of the tales of the Church in its younger and more enthusiastic days.

As Father Damien was a practical man, so did the Church Army seek for practical results, arising from the consideration of his life and work. After Mr. Clifford's address a resolution was proposed by Sir Donald Stewart, and seconded by Dr. Pringle, that the need for renewed investigation into the nature of leprosy is a pressing matter, and that Government should avail themselves of the offer of the Royal College of Physicians to take up the subject, especially in consideration of the welfare of our subjects in India. Dr. Pringle spoke very strongly on the necessity of segregation of lepers, and spoke with authority gained during many years' medical work in India. The Church Army desires to send forth labourers amongst the lepers of India, and one nurse has already volunteered for the service. In conclusion, we must state that Madame Antoinette Sterling, who is ever ready to aid a good cause, sang the twenty-third and thirty-seventh Psalms in her usual exquisite manner.

